# Supplementary figures and images for: Conventional dose rate spatially-fractionated radiation therapy (SFRT) treatment response and its association with dosimetric parameters—A preclinical study in a Fischer 344 rat model
Source: PLoS One. 2020 Jun 22;15(6):e0229053. doi: 10.1371/journal.pone.0229053 (PMC7307781; doi:10.1371/journal.pone.0229053)

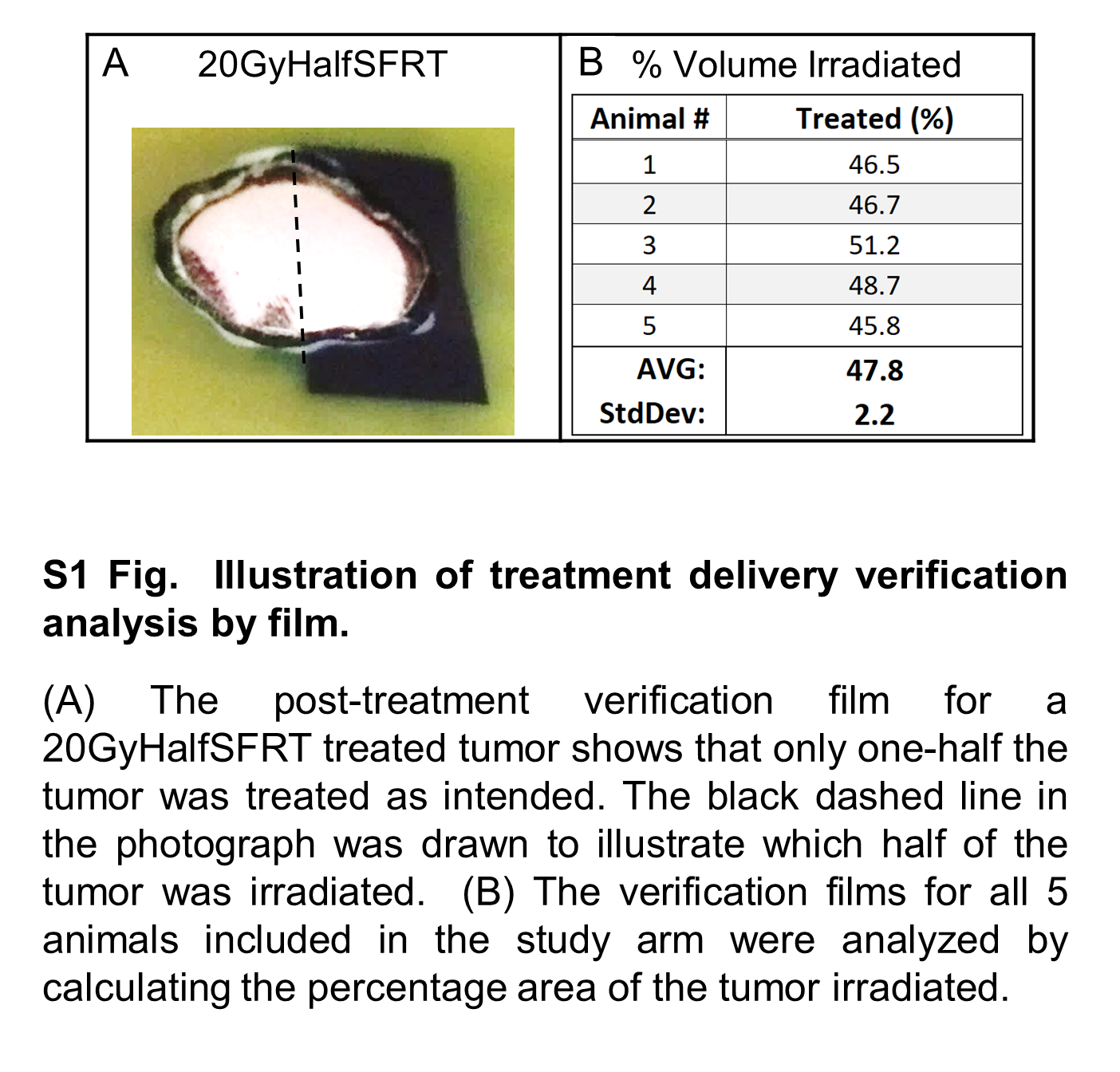

Supplement: S1 Fig — (A) The post-treatment verification film for a 20GyHalfSFRT treated tumor shows that only one-half the tumor was treated as intended. The black dashed line in the photograph was drawn to illustrate which half of the tumor was irradiated. (B) The verification films for all 5 animals included in the study arm were analyzed by calculating the percentage area of the tumor irradiated. (TIF) [file pone.0229053.s001.tif]

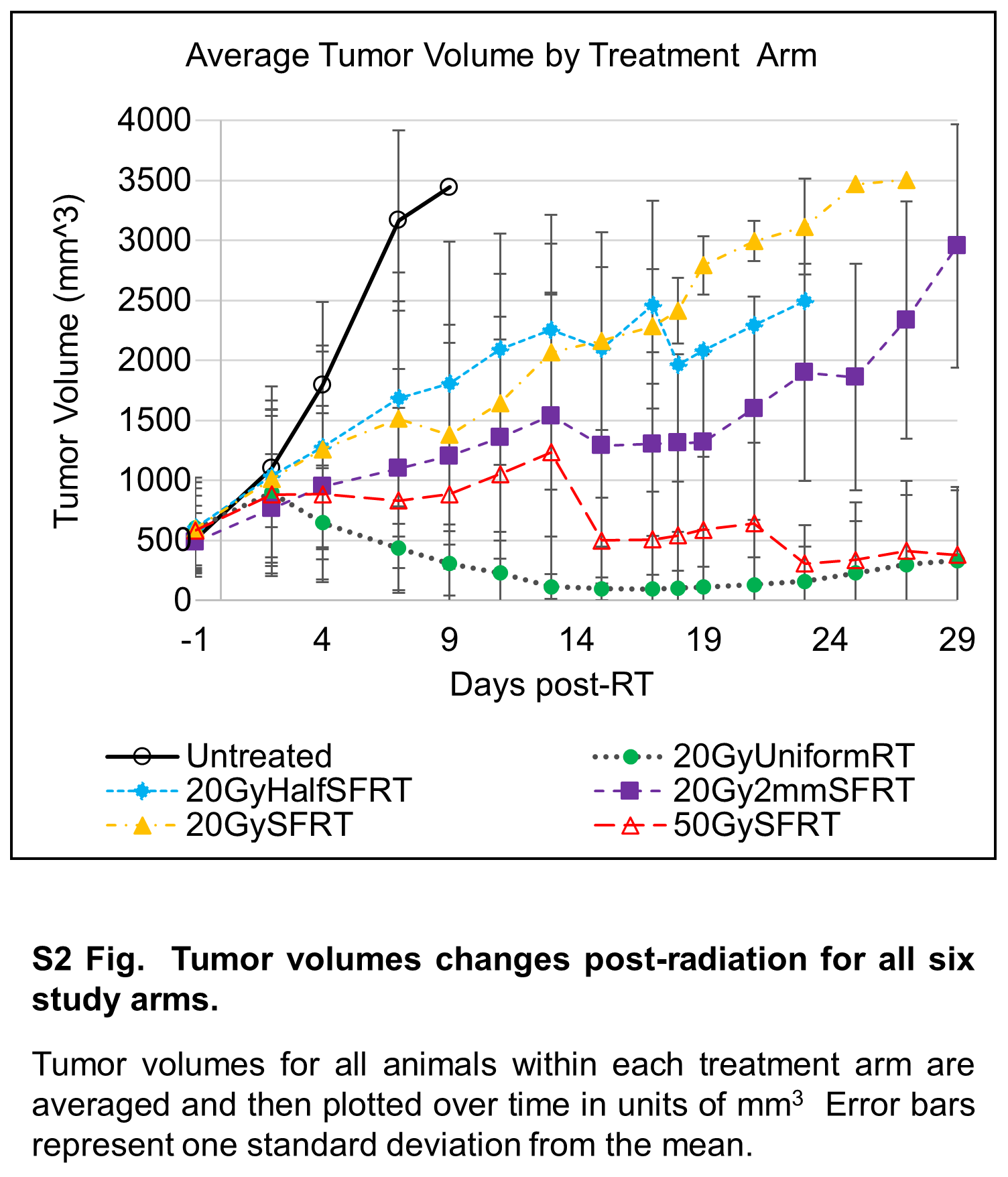

Supplement: S2 Fig — Tumor volumes for all animals within each treatment arm are averaged and then plotted over time in units of mm3. Error bars represent one standard deviation from the mean. (TIF) [file pone.0229053.s002.tif]

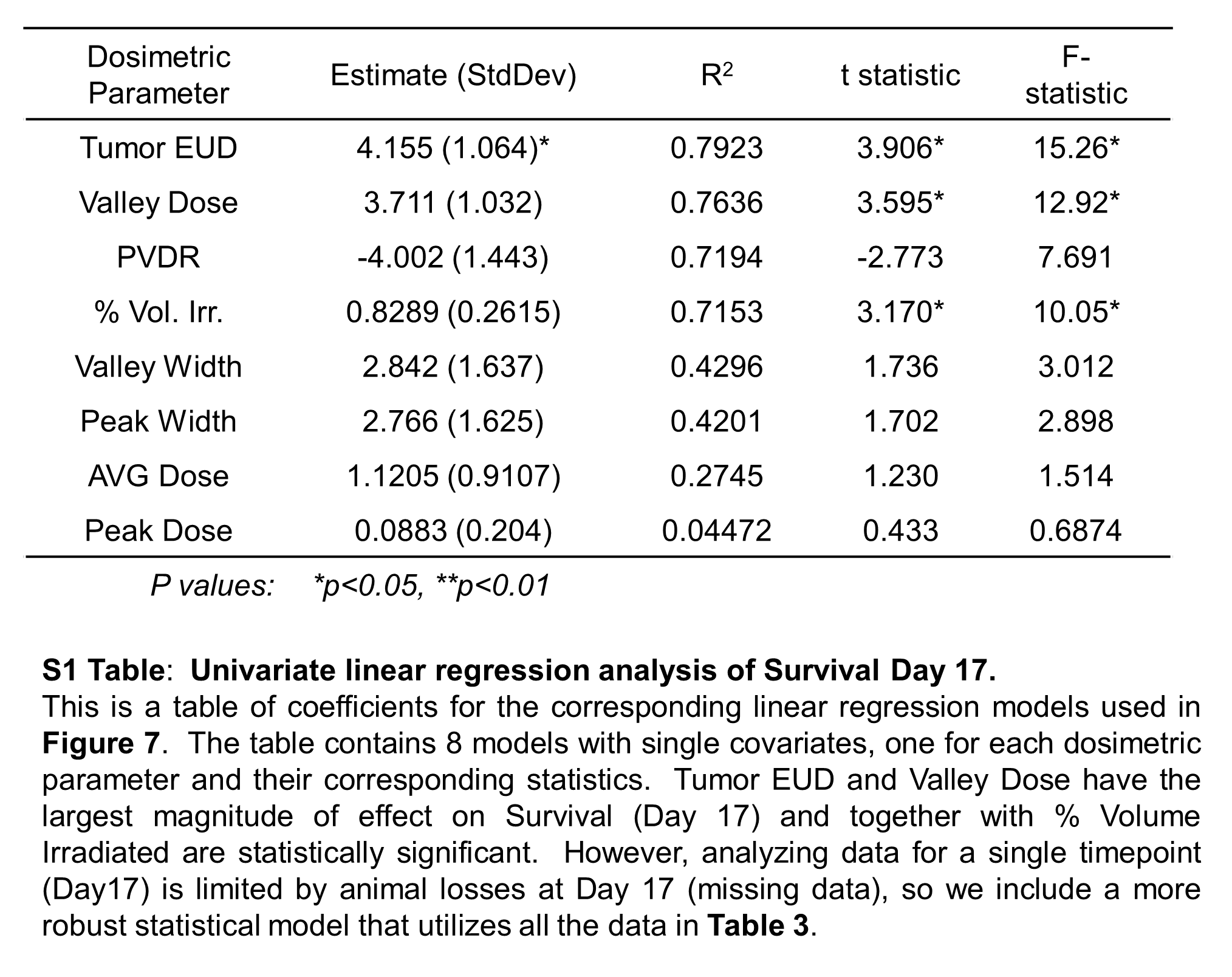

Supplement: S1 Table — This is the full table of coefficients for the corresponding linear regression models used in Fig 7. We analyze 8 models with single covariates, one for each dosimetric parameter and list their corresponding statistics. Tumor EUD and Valley Dose have the largest magnitude of effect on Survival (Day 17) and together with % Volume Irradiated are statistically significant. However, analyzing data for a single timepoint (Day17) is limited by animal losses at Day 17 (missing data), so we include a more robust statistical model that utilizes all the data in Table 4. (TIF) [file pone.0229053.s003.tif]

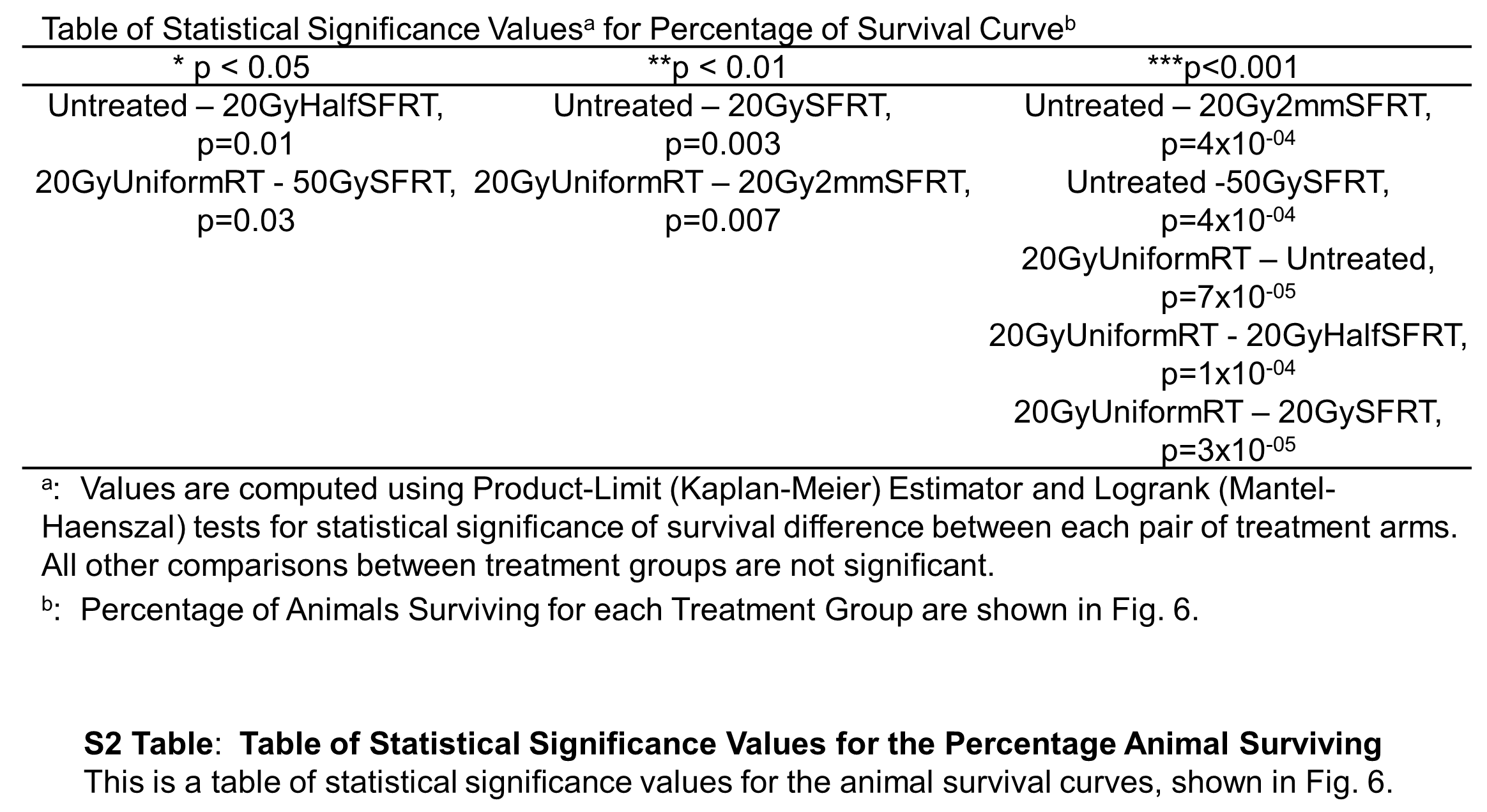

Supplement: S2 Table — This is a table of statistical significance values for the animal survival curves, shown in Fig 6. (TIF) [file pone.0229053.s004.tif]
